# Histamine H1 receptor: A target to treat pancreatic ductal adenocarcinoma (PDAC) by repurposing approved H1-antihistamines?

**DOI:** 10.46439/signaling.4.094

**Published:** 2026

**Authors:** Paul A. Insel, Cristina Salmerón, Mehrak Javadi-Paydar, Andrew M. Lowy, Peter McCormick

**Affiliations:** 1 Departments of Pharmacology and Medicine, University of California, San Diego, USA; 2 Department of Surgery, University of California, San Diego, USA; 3 Department of Medicine, University of California, San Diego, USA; 4 Department of Pharmacology, University of Liverpool, UK

**Keywords:** G protein-coupled receptors, Histamine, Histamine receptor 1, Pancreatic ductal adenocarcinoma, Antihistamines

## Abstract

New efficacious and safe therapies are needed for patients with pancreatic ductal adenocarcinoma (PDAC), the most common type of pancreatic cancer. A bioinformatic approach comparing human PDAC tumors to normal pancreas identified differentially expressed G protein-coupled receptors (GPCRs). The GPCRs with higher differential expression in PDAC included the histamine H1 receptor (HRH1), which, on average, is ~20-fold higher expressed in PDAC tumors. HRH1 is readily targetable by approved H1-antihistamines. Studies with human and murine PDAC tumor cells revealed that HRH1 agonists increase intracellular calcium in cancer cells and promote their migration and invasion. Fexofenadine, an H1-antihistamine, decreased pancreas tumor size and altered expression of cancer relevant genes in a mouse model of PDAC. These findings complement data for HRH1 and H1-antihistamines, especially second generation cationic amphiphilic antihistamines, observed in numerous other cancers. Studies are needed to determine if H1-antihistamines can augment anti-tumor response by current therapeutics and improve outcomes for PDAC patients. These findings with HRH1 suggest that other differentially expressed GPCRs with approved drugs may be therapeutic targets for PDAC and other cancers.

## Introduction

Much progress has been made in cancer diagnosis and treatment in recent years. New therapeutics, such as targeting cancer-specific mutations and relieving immune checkpoints, have increased patient survival of numerous types of cancer. However, not all cancer types have been successfully targeted by these new approaches. A prominent example is pancreatic ductal adenocarcinoma (PDAC, also termed pancreatic adenocarcinoma [PAAD]), for which targeted therapies and alteration of immune response have been largely ineffective (e.g., [1,2]). However, the recent development of allele-specific and pan-KRAS inhibitors holds promise, since most PDAC patients have KRAS mutations [3]. The 5-year survival for pancreatic cancer is 13% and for PDAC is 8% (American Cancer Society’s Cancer Statistics 2025). PDAC is currently the 3^rd^ leading cause of death from cancer in the United States and is predicted to become the 2^nd^ leading cause of cancer death (after lung cancer) by 2030 [4]. Thus, new effective and safe therapeutics are needed to treat PDAC patients.

G protein-coupled receptors (GPCRs) are the largest superfamily of membrane signaling proteins in multiple species. Humans have ~800 GPCRs, including 359 non-chemosensory (i.e., not mediating olfaction, taste or vision) receptors. GPCRs are also the largest class (~35%) of targets for currently approved drugs [5]. A recent review noted that more than 500 approved drugs target GPCRs, 337 agents target 133 GPCRs and 30 novel targets are currently in clinical trials [6].

With respect to cancers, GPCRs are targeted in endocrine gland tumors (e.g., pituitary, adrenal gland, thyroid, ovary and testis), certain neuroendocrine tumors (e.g., treated with somatostatin) and in non-endocrine tumors that respond to hormone receptor blockade (e.g., prostate cancer). However, GPCRs have generally not been considered as targets in other cancers, except for settings in which GPCRs or post-GPCR heterotrimeric G proteins are mutated (e.g., uveal melanoma and appendiceal cancers [7,8]). Interest is growing in the use of GPCRs as targets to improve immune responses, for example, involving chemokine receptors, such as CXCR4 (e.g., [9]) and perhaps other GPCRs [10].

## HRH1 Expression, Epidemiological Data and *In Vitro* Studies with PDAC Cancer Cells and Tumors

We hypothesized that GPCRs *without mutations* might be potential therapeutic targets if receptor expression was significantly higher in tumors compared to the normal tissue of origin. To test this idea, we used GPCRomics, an unbiased approach to identify and quantify the expression of most (using GPCR arrays, e.g., [11-13]) or all (RNA-sequencing [RNA-seq]) GPCRs (e.g., [14-17]) and bioinformatics to assess RNA-seq data [18]. Comparing the expression of GPCRs in 45 subtypes of solid tumors (The Cancer Genome Atlas [TCGA]) with healthy human tissues (Genotype-Tissue Expression [GTEx]) showed that most solid tumor subtypes have increased expression of >35 GPCRs (using criteria of at least 2-fold higher expression, more than 1 transcript per million and a false discovery rate of <0.05) [18]. Unexpectedly, >70 of the more highly expressed GPCRs in tumors are targeted by GPCR inhibitors approved by the United States Food and Drug Administration. Those drugs are potential candidates for repurposing as therapeutics for the cancers in which the GPCRs have higher differential expression.

PDAC has one of the largest number of GPCRs with higher differential expression and Histamine H1 receptor (HRH1), one of the 4 histamine receptors (the others being HRH2, HRH3 and HRH4 [19]), is one such GPCR [18]. Previous data suggest that multiple HRHs could influence the function of human PDAC cell lines (e.g., [20]). In 2020, Massari *et al.* reviewed histamine receptors and cancer pharmacology and emphasized “the pivotal role of HRH4 receptors in the development and progression of many types of cancers” along with HRH4’s immunomodulatory properties [21]. However, the differential expression of HRH1 in PDAC (comparing expression in GTEx and TCGA) did not reveal higher expression of the other 3 HRHs, as also shown in the Human Protein Atlas and other studies [22].

**Figure 1.**
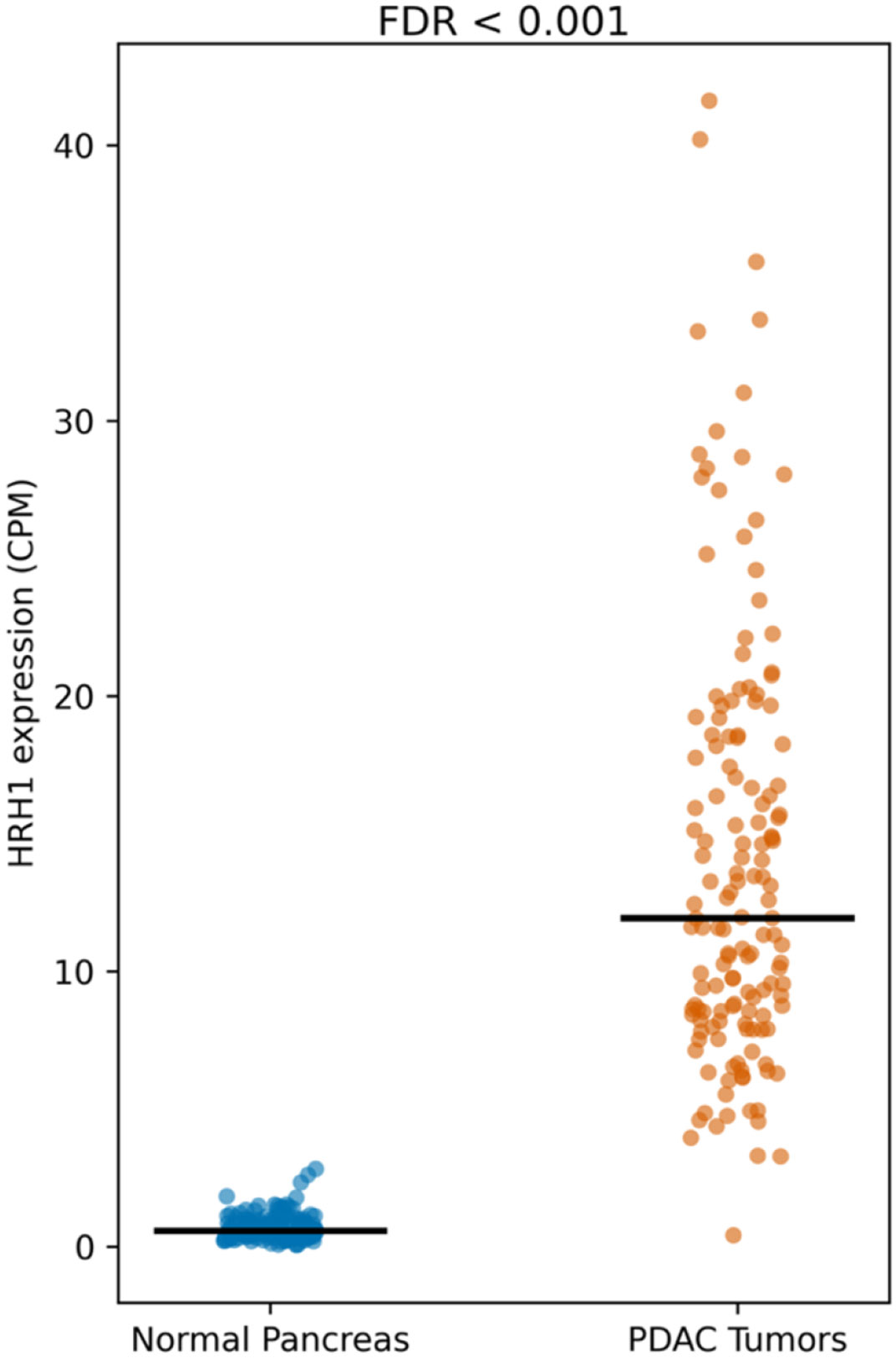
Comparison of the expression of HRH1 (from RNA-seq data) in TCGA-PDAC tumors (n=147 patients) and normal pancreas from GTEx (n=165 subjects). Overall, HRH1 expression is 20-fold greater in the PDAC tumors (Adapted from [23]).

Figure 1 shows the expression of HRH1 in patients with PDAC compared to subjects with a normal pancreas. PDAC patients with higher median expression of HRH1 have shorter survival than those with lower median HRH1 expression [23]. Expression of HRH1 in 14 human PDAC cell lines from the Cancer Cell Line Encyclopedia [24] is substantially higher than that of the other 3 HRHs, implying that PDAC cancer cells preferentially express HRH1. HRH1 is also more highly expressed in PDAC patient-derived organoids than in organoids from normal pancreas, further evidence that HRH1 is present in PDAC cancer cells [23].

Other studies suggest a role for HRH1 in PDAC. These include a retroactive study of a multiethnic cohort (white, African American, Latino, Japanese American and Native Hawaiian men and women) of patients with atopic allergic conditions who were treated with H1-antihistamines [25]. Older participants (age 70+) who used H1-antihistamines had a lower risk of developing pancreatic cancer (risk ratio 0.66, 95% confidence interval=0.45-0.96). Age 70 is the average age when pancreatic cancer is diagnosed (American Cancer Society, Pancreatic cancer risk factors 2025). Fritz and colleagues investigated the survival of ~429,000 Swedish patients with 10 types of cancers (including pancreatic cancer) who were taking H1-antihistamines (cetirizine, clemastine, desloratadine, ebastine, fexofenadine or loratadine) [26]. Desloratadine and loratadine improved the survival of certain cancers, including pancreatic cancer (hazard ratio 0.71, 95% confidence interval= 0.56–0.90). The authors speculated that the improved survival in numerous cancers derives from possible immunological actions mediated by H1-antihistamines.

We assessed protein expression, signaling and functional roles of HRH1 to validate findings from the RNA-seq data from human PDAC tumors and cells. We found that HRH1 protein (identified by immunofluorescence and immunohistochemistry) is present in cancer cells, macrophages and fibroblasts in PDAC tumors, as shown by colocalization with epithelial cell (E-Cadherin), macrophage (CD68) and α-smooth muscle actin markers, respectively [23].

HRH1 signals via Gq/11, which activate phospholipase C, and in turn, generates inositol trisphosphate and diacylglycerol, which respectively increase intracellular calcium (Ca^2+^) and activate protein kinase C [19,27]. In multiple human PDAC cell lines histamine and 2-pyridylethylamine dihydrochloride (2-PEA), an HRH1-selective agonist, increased intracellular Ca^2+^, a response blocked by H1-antihistamines. The EC_50_ values for the Ca^2+^ increase and the IC_50_/pKi for the H1-antihistamine fexofenadine (Allegra^®^) were similar to those observed in other cell types [19]. YM-254890, a Gq/11 inhibitor, blocked the increase in Ca^2+^ by histamine, implying that HRH1 response in PDAC cancer cells occurs via Gq/11.

Histamine and other GPCRs that increase Ca^2+^ promote migration in a wound-healing assay of human PDAC cancer cells, with response to histamine inhibited by H1-antihistamines [16,23]. Histamine and 2-PEA also increase (and fexofenadine inhibits) cell invasion in a hanging drop spheroid model [23]. Conditioned media from histamine-treated BxPC-3, a human PDAC cell line, increases VEGF-A, a mediator of blood vessel formation. Together, the signaling and functional studies indicate that HRH1 in human PDAC cancer cells has a “classical” HRH1 signaling mechanism and promotes responses that occur in malignancy [28].

## Studies of HRH1 in KPC Mouse Tumors and Cancer Cells

**Figure 2.**
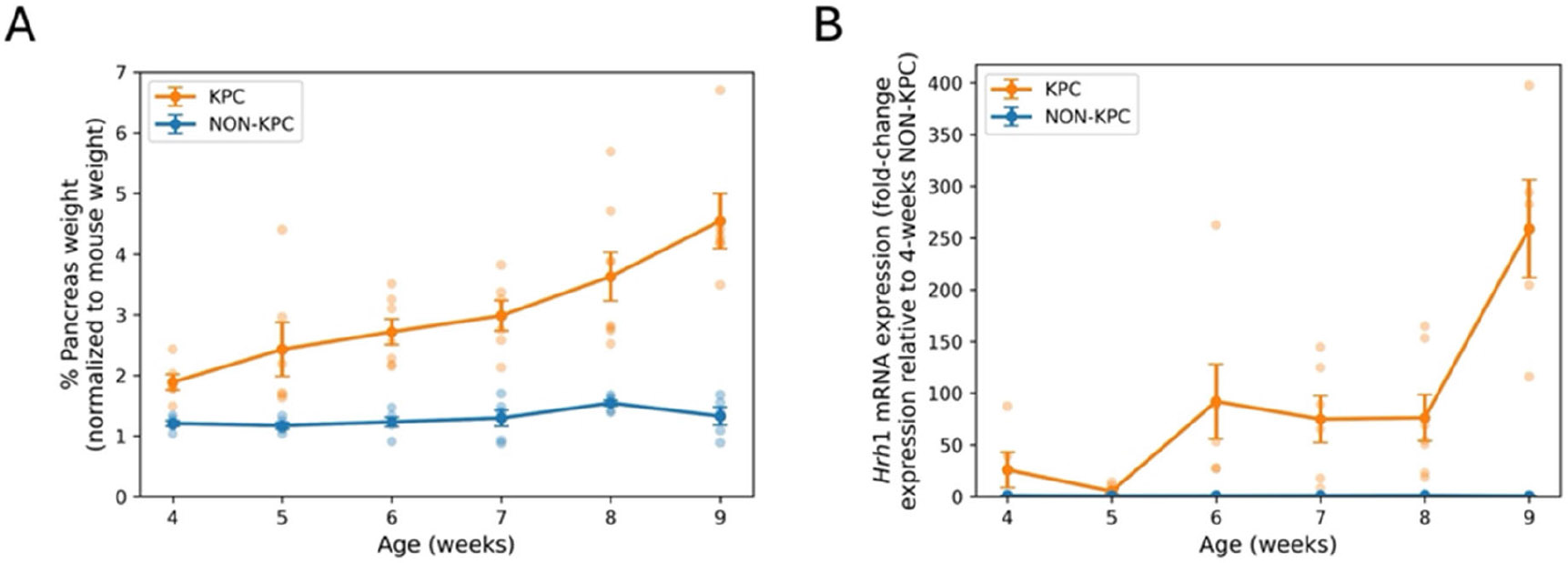
**(A)** Pancreatic weight (normalized to total body weight) in KPC and control (NON-KPC) mice from weeks-4 to 9. The data are shown as mean ± SEM (n=4–8) at each time point. The increase in pancreas weight in KPC mice results from their PDAC tumors. **(B)** HRH1 mRNA expression (using qPCR) in pancreatic tissue of KPC (n= 5–7) and control (NON-KPC) (n=3–6) mice from 4 to 9 weeks of age. The data are expressed as mean ± SEM (Adapted from [23]).

We studied a mouse model: KPC (Kras-LSL^G12D/+^; Trp53^fl/fl^; Ptf1a-Cre^+/−^) that spontaneously develops PDAC tumors histologically similar to human tumors [23]. Akin to human PDAC tumors, KPC tumors have higher expression of HRH1 mRNA (but not of other HRHs) and of HRH1 protein than in the normal pancreas of littermate controls. HRH1 expression in KPC-derived PDAC tumors increases during tumor growth (Figure 2). Moreover, histamine increased intracellular Ca^2+^ in a concentration-dependent manner in two cell lines generated from KPC tumors, a response blocked by fexofenadine [23]. Compared to non-KPC mice, KPC mice also have increased expression of mast cells, which synthesize and secrete histamine, as has been noted in mouse and human PDAC tumors (e.g., [20,29-32]).

To test if a H1-antihistamine might be an effective therapy for PDAC, we administered fexofenadine (or the solvent DMSO) to KPC mice in their drinking water, as previously used in mice [33], from age week 4 to week 9, when the mice were euthanized [23]. Fexofenadine treatment did not alter water consumption by the KPC mice and fexofenadine was present in PDAC tumors. We observed a 20.4% (p=0.036) decrease in pancreatic tissue weight and the tumors had decreased gene expression (compared to the DMSO controls) of the immunomodulator PD-1/*Pdcd1*, pro-inflammatory Interleukin-6/*IL-6*, and profibrotic Col 1a1/*Col1a1* [23], implying that histamine via HRH1 may impact on PDAC by effects in multiple cell types in the TME besides PDAC cancer cells.

The findings above show that HRH1 may contribute to the biology of PDAC tumors, at least in part via action in PDAC cancer cells. These data include; 1) epidemiological studies, 2) bioinformatic analyses that show substantially higher expression of HRH1 in PDAC tumors than in the normal pancreas, 3) HRH1 mRNA and protein expression in several PDAC cell types, 4) signaling by HRH1 in PDAC tumor cells that is similar to that of HRH1 in other cell types, 5) functional activity of HRH1 in PDAC cells with similar findings in KPC and human PDAC cells, and 6) the ability of an H1-antihistamine to decrease pancreas tumor growth along with expression of PDAC cancer relevant genes. Thus, H1-antihistamines may be an approach to treat patients with PDAC.

## Discussion: An Emphasis on Cationic Amphiphilic H1-Antihistamines and HRH1 in Other Cancers

Further studies related to HRH1 and PDAC might involve the following: 1) *in vitro* mechanistic assessment of the impact of HRH1 activation on additional aspects of PDAC biology; 2) Comparison of efficacy of different types of H1-antihistamines (e.g., cationic vs. non-cationic amphiphilic agents, as discussed below) in blunting actions of histamine in PDAC models; 3) *in vitro* experiments that assess impact of H1-antihistamines together with standard of care agents used to treat PDAC patients (e.g., gemcitabine), immune therapy (e.g., [34]), and newer approaches (e.g., pan-KRAS inhibitors [3]); 4) studies in human tissue models, such as PDAC cell organoids and organotypic tissue slices [35]; 5) additional *in vivo* studies with KPC (or other PDAC mouse models) that test other H1-antihistamines (perhaps comparison of cationic and non-cationic amphiphilic H1-antihistamines) together with other therapeutic approaches; (our *in vivo* studies thus far used fexofenadine [23], a non-cationic amphiphilic H1-antihistamine); and 6) further retrospective studies of PDAC patients who did or did not use H1-antihistamines from additional countries than those previously studied.

In line with our findings, Zhong *et al.* [22] reported that the expression of HRH1 in PDAC cancer cells is negatively correlated with HLA-ABC expression, CD8^+^ T cells, and cytotoxic CD8^+^ T cells. The authors observed that H1-antihistamines increase MHC-1 expression in PDAC cancer cells by cholesterol biosynthesis signaling. The combination of inhibition of HRH1 and αPD-1 increased the action of CD8^+^ T cells and helped overcome resistance to immune checkpoint therapy, thus potentially offering an approach to enhance the efficacy of immunotherapy in PDAC. This study also provided evidence implying that an H1-antihistamine (azelastine, a cationic amphiphilic antihistamine) could reverse the ability of cancer associated fibroblasts to inhibit aspects of immunotherapy responses.

Several studies have emphasized the advantage of second-generation cationic amphiphilic (e.g., loratadine, desloratadine, azelastine, clemastine, astemizole) rather than non-cationic amphiphilic H1-antihistamines for the treatment of cancers. First-generation H1-antihistamines (e.g., diphenhydramine [Benadryl^®^]) have side effects, in particular drowsiness, which is generally not present in second-generation H1-antihistamines. Cationic amphiphilic antihistamines have a hydrophobic (lipophilic) aromatic region plus a strongly basic amine side chain (positive charge at pH 7.4) that become protonated and are thought to accumulate in lysosomes, which may induce cancer cell death via lysosomal membrane permeabilization, release of Ca^2+^ and perhaps cAMP [36,37]. Another possible mechanism is by antihistamine-mediated neutralization of the negative charge of lysosomal lipases, in particular acid sphingomyelinase, thereby increasing sphingomyelin, which is toxic to cancer cells [38]. By contrast, non-cationic amphiphilic antihistamines are zwitterionic (having equal number of positive and negative charges) at physiologic pH and include drugs such as fexofenadine and cetirizine (Zyrtec^®^).

Second-generation cationic amphiphilic antihistamines have been shown to improve the survival of cancer patients who receive immune checkpoint inhibitors [39] and in a small group of PDAC patients in Taiwan who were users of cationic amphiphilic antihistamines (n=28) or of non-cationic amphiphilic antihistamines (n=56) [40]. Another study showed the key role of HRH1-activated macrophages in producing T cell dysfunction, which was blunted with H1-antihistamines [41]. The authors also noted that cancer patients with low plasma histamine levels had a greater response to aPD-1 treatment than those with high levels, particularly in lung, breast, and colon cancers. Experiments with mice in that study involved the use of fexofenadine and showed positive effects on aspects of tumor biology.

A cohort study of Danish women with ovarian cancer found that women taking cationic amphiphilic antihistamines had better survival (hazard ratio 0.63; 95% confidence interval = 0.40-0.99) whereas no such association was observed for non-cationic amphiphilic antihistamines [42]. A study of Danish patients with non-localized non-small cell lung cancer also revealed a decreased all-cause mortality in those using cationic amphiphilic antihistamines compared to those using non-cationic antihistamines with a hazard ratio and confidence intervals for loratadine similar to what was observed in the study of women with ovarian cancer [43]. As noted above, it has been suggested that antihistamines may enhance immunotherapy of cancers (e.g., [34]). Two recent reviews of several studies concluded that cancer patients using H1-antihistamines together with immunotherapy had longer median overall survival, progression-free survival, or improved survival than did those who did not use antihistamines [44,45]. Both reviews noted that patients who used cationic amphiphilic H1-antihistamines had better median overall survival and progressive-free survival than patients who used non-cationic amphiphilic H1-antihistamines.

Fritz *et al.*, who assessed Swedish patients, showed improved survival in breast cancer in patients who use H1-antihistamines, particularly cationic amphiphilic H1-antihistamines [46]. Another retrospective analysis concluded that antihistamines may reduce the risk of certain cancers [47]. In recent years, other studies have observed a decreased risk for cancer, or assessed expression and/or functional activity of histamine and HRH1 or H1-antihistamines in multiple types of cancer other than PDAC [41,47-49], including hepatocellular carcinoma (e.g., [50-52]), glioblastoma (e.g., [53-55]), oral squamous cell carcinoma [56], cholangiocarcinoma [57], breast cancer [58], bladder cancer [59], melanoma [41], osteosarcoma [60], and colon cancer [61]. Other studies have implicated a role for histamine and HRH1 in promoting angiogenesis in tumors (e.g., [53,62]).

## Conclusions

In summary, a growing body of evidence suggests that histamine, acting via HRH1, can contribute to the biology of multiple cancers, including PDAC. Results from numerous studies, including retrospective analyses of patients, imply that second-generation cationic amphiphilic H1-antihistamines can improve the survival and outcome, including perhaps for PDAC patients (e.g., [22,40,44]). Given the need for new, safe and efficacious treatments for PDAC, we and our colleagues have proposed that a prospective clinical trial should be undertaken to determine if a second-generation cationic amphiphilic H1-antihistamine can improve the survival and overall clinical course of PDAC patients, since such drugs have well defined pharmacokinetics and side-effects (which are generally mild) [63]. A clinical trial would likely include the current standard of care with and without a second-generation cationic amphiphilic H1-antihistamine to test efficacy and safety of the antihistamine. Perhaps H1-antihistamines will enhance the action of immunotherapy [44,45], which previously has been ineffective in the treatment of PDAC [1,2,64,65].

From a more general perspective, beyond the potential utility of H1-antihistamines to treat PDAC and perhaps other cancers, numerous types of cancer have enhanced expression of GPCRs (compared to GPCR expression in their tissue of origin [18]), some of which have approved drugs that could be repurposed. Studies that assess such GPCRs in other types of cancer have the potential to yield new therapeutic approaches, most likely in combination with other therapies, with the goal of improving the survival and outcome of cancer patients.

## Abbreviations:

aPD-1: Anti-PD1 Antibody
Ca^2+^: Calcium
DMSO: Dimethyl Sulfoxide
GPCRs: G Protein-Coupled Receptors
Gq/11: Heterotrimeric GTP Binding Proteins q/11
GTEx: Genotype-Tissue Expression
HRH: Histamine Receptor
MHC: Major Histocompatibility Complex
PAAD: Pancreatic Adenocarcinoma
PDAC: Pancreatic Ductal Adenocarcinoma
PD-1/*Pdcd1*: Programmed Cell Death Protein 1
RNA-seq: RNA-sequencing
TCGA: The Cancer Genome Atlas
2-PEA: 2-Pyridylethylamine Dihydrochloride
TME: Tumor Microenvironment
VEGF-A: Vascular Endothelial Growth Factor-A
